# HDAC3 mediates smoking-induced pancreatic cancer

**DOI:** 10.18632/oncotarget.6820

**Published:** 2016-01-05

**Authors:** Mouad Edderkaoui, Shiping Xu, Chintan Chheda, Susan Morvaridi, Robert W. Hu, Paul J. Grippo, Emman Mascariñas, Daniel R. Principe, Beatrice Knudsen, Jing Xue, Aida Habtezion, Dale Uyeminami, Kent E. Pinkerton, Stephen J. Pandol

**Affiliations:** ^1^ Departments of Medicine and Biological Sciences, Cedars-Sinai Medical Center, Los Angeles, CA, USA; ^2^ Veterans Affairs Greater Los Angeles Healthcare System & University of California at Los Angeles, CA, USA; ^3^ Department of Gastroenterology, Nanlou Division, The PLA General Hospital, Beijing, China; ^4^ Robert H. Lurie Comprehensive Cancer Center, Feinberg School of Medicine, Northwestern University, Chicago, IL, USA; ^5^ Department of Medicine, University of Illinois-Chicago, Chicago, IL, USA; ^6^ Division of Gastroenterology and Hepatology, Department of Medicine, Stanford University School of Medicine, Stanford, CA, USA; ^7^ Center for Health and the Environment, University of California, Davis, CA, USA

**Keywords:** smoking, HDAC, pancreatic cancer

## Abstract

Smoking is a major risk factor for developing pancreatic adenocarcinoma (PDAC); however, little is known about the mechanisms involved.

Here we employed a genetic animal model of early stages of PDAC that overexpresses oncogenic Kras in the pancreas to investigate the mechanisms of smoking-induced promotion of the disease *in vivo*. We confirmed the regulation of the interactions between the tumor microenvironment cells using *in vitro* cellular systems.

Aerial exposure to cigarette smoke stimulated development of pancreatic intraepithelial neaoplasia (PanIN) lesions associated with a tumor microenvironment-containing features of human PDAC including fibrosis, activated stellate cells, M2-macrophages and markers of epithelial-mesenchymal transition (EMT). The pro-cancer effects of smoking were prevented by Histone Deacetylase HDAC I/II inhibitor Saha.

Smoking decreased histone acetylation associated with recruitment of and phenotypic changes in macrophages; which in turn, stimulated survival and induction of EMT of the pre-cancer and cancer cells. The interaction between the cancer cells and macrophages is mediated by IL-6 produced under the regulation of HDAC3 translocation to the nucleus in the cancer cells. Pharmacological and molecular inhibitions of HDAC3 decreased IL-6 levels in cancer cells. IL-6 stimulated the macrophage phenotype change through regulation of the IL-4 receptor level of the macrophage.

This study demonstrates a novel pathway of interaction between cancer cells and tumor promoting macrophages involving HDAC3 and IL-6. It further demonstrates that targeting HDAC3 prevents progression of the disease and could provide a strategy for treating the disease considering that the HDAC inhibitor we used is FDA approved for a different disease.

## INTRODUCTION

Pancreatic ductal adenocarcinoma (PDAC) is the fourth leading cause of cancer-related deaths among both men and women in the United States and remains without effective therapies [1, 2]. Environmental risk factors such as smoking, diabetes, obesity and alcoholism play major roles in the promotion of PDAC [3-5]. However, we have little understanding of how these risk factors promote the disease. Animal models expressing genetic changes observed in pancreatic cancer have been developed and have greatly improved our understanding of the disease. However, models incorporating these risk factors have been lacking. Because cigarette smoking is an established and major risk factor for PDAC [6, 7], models designed to determine the mechanisms of smoking-induced PDAC can provide important insights into the promotion of this cancer.

HDAC enzymes are critical regulators of fundamental cellular events such as cell cycle, differentiation, and apoptosis [8]. HDAC1 has been shown to be involved in regulating epithelial to mesenchymal transition (EMT) in pancreatic cancer cells by directly interacting and stabilizing the pro-EMT Zeb1 transcription factor [9]. EMT is considered a critical process of cell transformation in cancer metastasis. In addition to PDAC, risk factors such as smoking and alcohol abuse cause inflammatory diseases of the pancreas such as acute and chronic pancreatitis. Previous reports indicate a key role for pancreatic inflammation in the promotion of PDAC in both humans and animal models. In addition, there is increasing evidence that sub-types of macrophages play a major role in promotion of many cancers including pancreatic cancer [10, 11]. There is a strong correlation between M2 type macrophages and increased metastasis and poor prognosis in humans with PDAC [12].

Little is known about the intracellular mechanisms involved in secretion of cytokines from early cancer lesions such as pancreatic intraepithelial neoplasia (PanIN) and cystic papillary neoplasia (CPN similar to human IPMN and MCN) and cancer cells that are in communication with cells in the tumor microenvironment. Data presented here provide evidence for a role of HDAC in regulating cytokine secretion involved in promotion of the pro-tumor type-2 macrophage (M2) phenotype and tumor promotion. That is, inhibition of HDAC specifically inhibited interleukin 6 (IL-6) production by cancer cells that we found was necessary for the macrophage phenotype change in our animal model of PDAC. These results point to roles for HDAC and IL-6 in regulation of macrophages by neoplastic lesions and cancer cells.

It has been shown that IL-6 is able to induce interleukin-4 (IL-4) production in T cells, thereby polarizing these cells into Th2 cells [13, 14]. Very little is known about the effect of IL-6 on macrophages. Our data show a role of IL-6 secreted by the cancer cells on inducing the M2 macrophage phenotype.

Multiple human case-control studies have linked high serum levels of IL-6 with pancreatic cancer and suggested using it as a marker of cancer progression. A strong positive correlation was found among tumor stage, cachexia and decreased survival [15-20].

In the present study we developed two mouse models of pancreatic cancer precursors where Pdx1-Cre;LSL-Kras (KC) mice were exposed to cigarette smoke in smoke chambers. Exposure to cigarette smoke stimulated pancreatic neoplasia (PanIN in KC mice) formation associated with a tumor microenvironment containing features of human PDAC including fibrosis, activated stellate cells, M2 macrophages along with markers of epithelial to mesenchymal transition (EMT) and cancer stemness. We found that HDAC3 plays a major role in mediating these smoking pro-cancer effects and that inhibition of HDAC3 prevents these effects. Our work goes on to demonstrate that HDAC3 specifically regulates IL-6 production in cancer cells in the tumor microenvironment; and that IL-6 is involved in regulating macrophage function including the promotion of the M2 phenotype.

## RESULTS

### Cigarette smoke stimulates PanIN lesions formation in KC mice and HDAC inhibition reverses this effect

**Figure 1:**
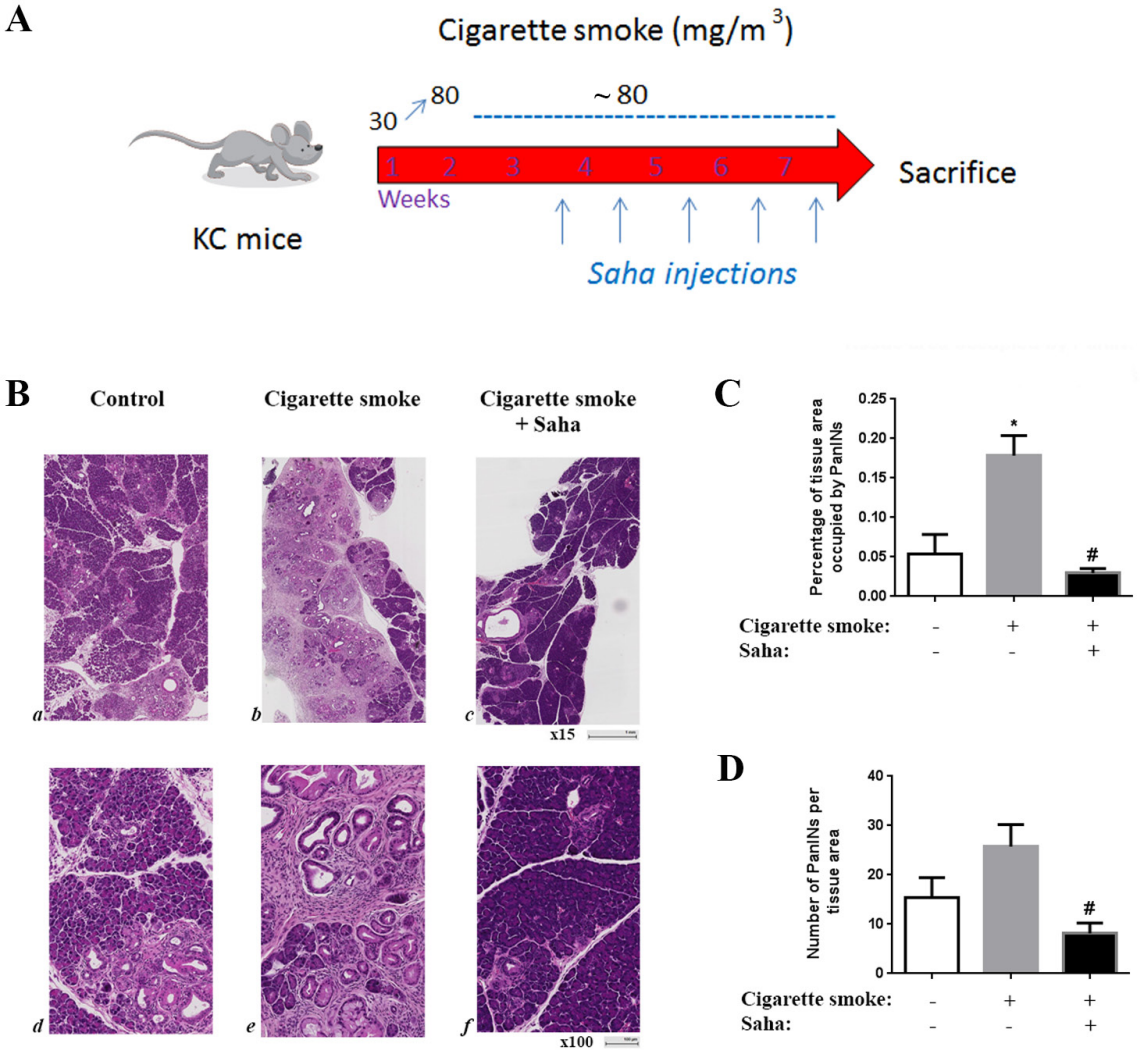
Cigarette smoke exposure stimulates pancreatic lesion formation and HDAC inhibition prevents this effect in KC mice KC mice were exposed to cigarette smoke or filtered air for 7 weeks and injected with saline or Saha (50mg/Kg) 5 days a week during the last 5 weeks **A**. **B.** H&E staining of the pancreas of the KC mice exposed to filtered air (a, d) or cigarette smoke (b, e) or cigarette smoke with injections of HDAC inhibitor Saha (c, f). **C.** Quantification of tissue area occupied by PanIN lesions. **D.** Quantification of the number of PanIN lesions per tissue area. *p < 0.05 *versus* control. #*p* < 0.05 *versus* cigarette smoke.

We exposed KC mice to cigarette smoke for 7 weeks and treated mice with the HDAC inhibitor Saha. The KC mice carry the Kras mutation in the pancreas and spontaneously develop PanIN lesions, considered to be precursors of PDAC [21] Kras mutations are present in 90% of pancreatic cancer patients [22, 23]. KC mice recapitulate the characteristics of the human disease including a vast desmoplastic reaction with rapid growth, and represent the early stage of the disease [21]. KC mice were exposed to cigarette smoke for 7 weeks and injected with Saha or saline during the last 5 weeks of the treatment (Fig 1A). During the 7 weeks of smoking exposure, levels of carbon monoxide and gravimetric total smoke particles (TSP) were measured every day and the levels of nicotine measured every week (Table 1). TSP was increased during the first week gradually to reach 80mg/m^3^.

**Table 1:** Measurements of Carbon Monoxide (CO) and total smoke particles (TSP)

	Carbon Monoxide (ppm)	CO StdDev (ppm)	Nicotine (mg/m3)	Gravimetric TSP (mg/m3)	TSP StdDev (mg/m3)
Day 1	157.73	11.04		41.31	2.29
Day 2	172.50	11.37		50.55	3.36
Day 3	217.27	7.86	12.10	65.76	3.26
Day 4	216.36	11.42		63.77	7.60
Day 5	230.91	18.41		70.47	7.75
Day 8	243.64	8.69		75.00	6.05
Day 9	240.00	10.00		73.67	2.55
Day 10	264.09	5.84	13.70	83.58	3.02
Day 11	263.33	6.61		82.61	6.15
Day 12	265.91	13.75		85.51	7.67
Day 15	255.00	11.55		83.57	3.99
Day 16	229.09	8.01		75.12	3.43
Day 17	166.25	9.16		48.31	6.33
Day 18	167.14	3.93		50.48	2.33
Day 19	188.64	8.39	9.40	56.28	2.74
Day 22	230.00	9.57		63.77	3.84
Day 23	205.71	14.84		64.73	5.44
Day 24	213.18	6.03		71.38	2.81
Day 25	214.44	3.91	10.40	71.01	1.45
Day 26	211.82	5.13		73.43	3.02
Day 29	215.00	2.89		68.36	7.69
Day 30	213.64	3.23		66.36	1.67
Day 31	216.67	6.12		66.67	2.62
Day 32	233.64	7.45	8.80	80.44	8.17
Day 33	235.56	11.30		74.16	5.81
Day 36	231.36	8.97		71.74	2.51
Day 37	234.00	15.78	11.90	62.56	3.58
Day 38	244.09	16.40		69.56	5.07
Day 39	237.73	6.84	11.90	82.97	4.68
Day 40	232.73	5.18		71.50	7.26
Day 43	252.78	11.76		65.94	6.91
Day 44	239.55	8.50	9.70	81.64	5.86
Day 45	238.33	5.59		82.61	4.75
Day 46	247.50	6.12		76.54	6.79
Day 47	256.67	4.08		73.14	6.22
**Mean:**	**225.21**	**8.73**	**10.86**	**70.11**	**4.7**
**StDev**	**27.87**	**3.91**	**1.76**	**10.82**	**2.04**

Analysis of the pancreatic tissue from KC mice exposed to cigarette smoke showed a marked increase in the number and tissue area occupied by PanIN lesions compared to control treated mice. The tissue area containing PanINs was increased by over 300% and the number of lesions was increased by ~70% (Fig 1B-1D). More importantly, this increase was abolished when mice were exposed to HDAC inhibitor Saha. The number and tissue area covered by PanINs was decreased to a level less than in control mice suggesting that HDAC not only mediated the smoking effect but also contributed in the basal progression of the disease (Fig 1C, 1D).

**Figure 2:**
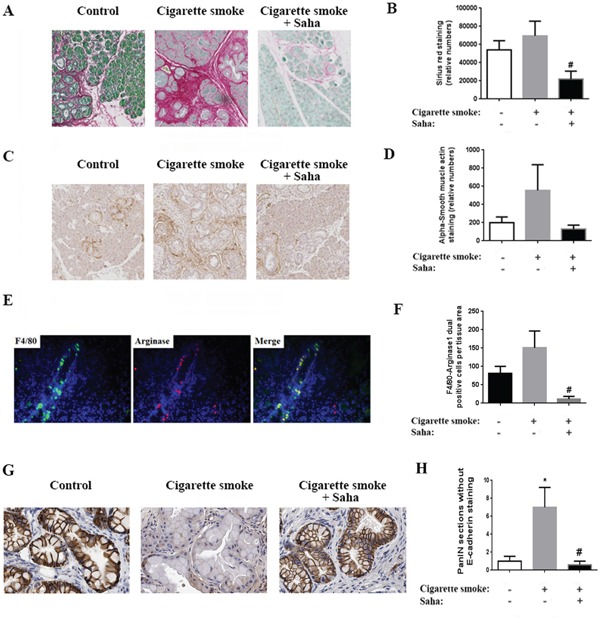
Cigarette smoke exposure stimulates fibrosis, macrophage M2 phenotype induction and EMT in the pancreas of KC mice **A.** Sirius Red/Collagen staining of the pancreas of KC mice exposed to filtered air or cigarette smoke with or without HDAC inhibitor Saha. **C.** α-SMA staining of mice pancreas. **E.** Macrophage dual staining with F4/80 and Arginase1. **G.** E-cadherin staining of mice pancreas. (B, D, F, H) Quantification of the staining in the right of each panel. *p < 0.05 *versus* control. #*p* < 0.05 *versus* cigarette smoke.

Other characteristics of pancreatic cancer such as fibrosis and stellate cells activation were stimulated by cigarette smoke, and prevented by Saha (Fig 2A, 2B). Collagen staining showed an increase of 35% and α-SMA by 2.5 times. Saha reversed these effects decreasing collagen and α-SMA level by 70% (Fig 2A, 2B).

Furthermore, dual staining of macrophages by the general marker F4/80 and M2 macrophage marker Arginase1 showed a 30% increase in the M2 macrophages in the pancreas of mice exposed to cigarette smoke (Fig 2E). The increase in the number of M2 macrophages was nearly abolished by Saha and was decreased by almost 90% (Fig 2F). In addition, we found that smoking stimulated EMT in mice pancreas as shown by E-cadherin staining of the pancreatic tissue. This effect was inhibited with Saha (Fig 2G).

In sum, the results above indicate that exposure to cigarette smoke stimulates PanIN lesion formation, fibrosis and inflammation, especially the presence of M2 macrophages, and EMT. More importantly, the administration of Saha to inhibit HDAC prevented these pro-cancer effects of smoking.

### Interaction between PanIN/cancer cells and M2 macrophages mediates promotion of pancreatic cancer

**Figure 3:**
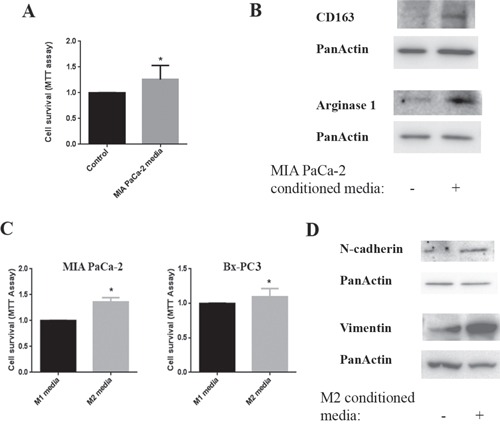
Interaction between cancer cells and macrophages Cells were cultured for 24h and then conditioned media applied to the other cell type for 24h. **A.** Cell survival of macrophages cultured in MIA PaCa-2 conditioned media measured by MTT assay. **B.** Protein levels of CD163 and Arginase1were measured by Western in the lysates of macrophages cultured in MIA PaCa-2 conditioned media; Blots were re-probed for PanActin to confirm equal loading. **C.** Cell survival of MIA PaCa-2 and Bx-PC3 cells cultured in M1 and M2 macrophage conditioned media measured by MTT assay. **D.** Protein levels of N-cadherin and vimentin were measured by Western in lysates of MIA PaCa-2 cells cultured in M0/M1 or M2 macrophage conditioned media; blots were re-probed for PanActin to confirm equal loading. *p < 0.05 *versus* control.

Strong evidence shows a pro-cancer role of M2 macrophages in tumor development and worse outcome in pancreatic cancer patients [24]. Therefore, we established studies to analyze the potential interactions between macrophages and pancreatic cancer cells. We found that the pancreatic cancer cell line MIA PaCa-2 conditioned media increased the survival of macrophages (Fig 3A) and more importantly stimulated a phenotype change of the macrophages towards the M2 phenotype as measured by its markers CD163 and Arginase1 (Fig 3B). Next, we cultured pancreatic cancer cell lines in the presence of macrophage conditioned media and found that M2 macrophage conditioned media promoted survival of both MIA PaCa-2 and Bx-PC3 cells compared to the M1 macrophage conditioned media (Fig 3C). In addition, M2 macrophage conditioned media stimulated EMT in pancreatic cancer cells as shown by measuring the level of the EMT markers N-cadherin and vimentin (Fig 3D). Of note, E-cadherin, N-cadherin, and vimentin measurements we used in the previous figures are established markers of EMT [25].

### p-HDAC3 and IL-6 mediate the interaction between cancer cells and macrophages

The results in Fig 3 led us to investigate the composition of the cancer cell conditioned media to determine what mediates the change in macrophage phenotype induced by the cancer cells.

**Figure 4:**
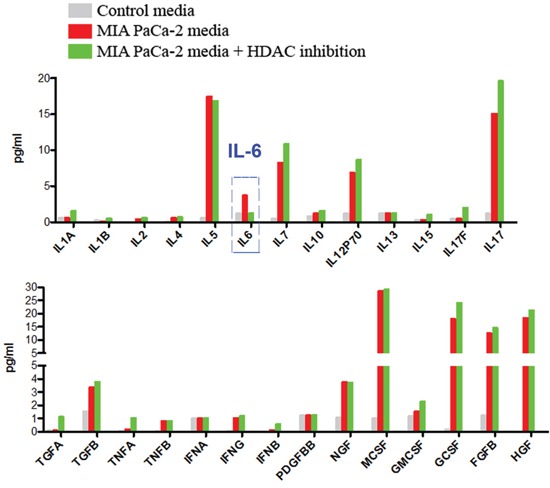
Multiplexing analysis of the secretions of pancreatic cancer cells **A.** Multiplexing analysis of growth factors, cytokines and chemokines expressed and secreted in the media by MIA PaCa-2 cells cultured for 24h in the presence of absence of HDAC inhibitor Saha. Data is representative of 3 different measurements that give similar results.

Therefore, we analyzed the growth factors and cytokines secreted by cancer cells in the presence and absence of HDAC inhibition using multiplex analysis. We found that Saha did not affect production of any of the analytes except the cytokine IL-6, suggesting that IL-6 mediates the regulation of the macrophage phenotype by cancer cells (Fig 4).

**Figure 5:**
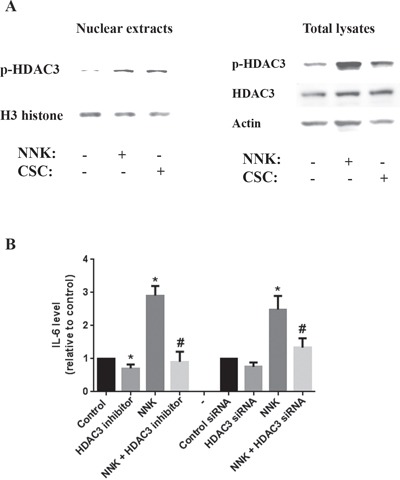
Smoking stimulates IL-6 production through a mechanism that involves HDAC3 translocation to the nucleus MIA PaCa-2 cells were incubated for 1h in the presence or absence of smoking compounds NNK and CSC or in the presence of HDAC3 specific inhibitor RGFP966 (1μM). **A.** Protein level of p-HDAC3 in nuclear extracts and total lysates is measured by Western. Blots were re-probed for H3 histone or GAPDH to confirm equal loading. **B.** Cells were cultured with NNK (1 μM) for 48h and IL-6 level measured by multiplexing. MIA PaCa-2 cells were transfected with scrambled or HDAC3 siRNA using the electroporation Amaxa System Nucleofector according to the manufacturer protocol. *p < 0.05 *versus* control. #*p* < 0.05 *versus* NNK.

To determine which member of the HDAC families I and II (inhibited by saha) is involved in regulating IL-6 production we analyzed the protein level and localization of members of both families. We found that HDAC3 phosphorylation level as well as the level of p-HDAC3 in nuclear extracts were significantly increased by both NNK and Cigarette smoke Condensate (CSC) smoking compounds (Fig 5A). The level of total HDAC3 in total lysates did not change (Fig 5A) indicating that smoking compounds affect only the phosphorylation status and the translocation of HDAC3 to the nucleus but not its protein level.

Next, we measured the effect of smoking compounds on secretion of IL-6 by the cancer cells. We found that NNK increased the level of IL-6 in MIA PaCa-2 conditioned media by up to 3 folds (Fig 5B). This effect was abolished when applying a pharmacological or molecular inhibition of HDAC3 (Fig 5B).

**Figure 6:**
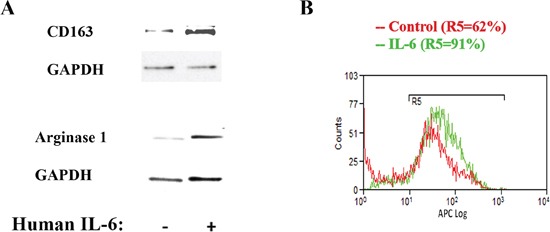
IL-6 up-regulates macrophage M2 phenotype and stimulates IL-4 receptor expression **A.** Human macrophages were cultured in the presence of IL-6 (20 ng/ml) for 24h and the level of Arginase1 and Cd163 measured by Western. Blots were re-probed for GAPDH to confirm equal loading. **B.** Human macrophages were cultured in the presence of IL-6 (20 ng/ml) for 24h and the level of IL-4 receptor measured by flow cytometry.

To confirm the regulation of macrophage phenotype by IL-6 we added IL-6 to the macrophages and found that IL-6 increased the M2 phenotype as measured by the level of two M2 macrophages markers Arginase1 and CD163 consistent with the *in vitro* and *in vivo* results described above (Fig 6A). One mechanism through which IL-6 may mediate macrophage phenotypic change is through up-regulation of the IL-4 receptor level [26]. Results in Fig 6B show that exposure of human macrophages, pre-incubated for 24h with IL-4, to recombinant IL-6, indeed, increases the number of cells expressing IL-4 receptor from 62% to 91% of the total population of macrophages (Fig 6B).

### p-HDAC3, IL-6, M2 macrophages, and IL-4 receptor were highly expressed and histone acetylation decreased in human pancreatic tumors

**Figure 7:**
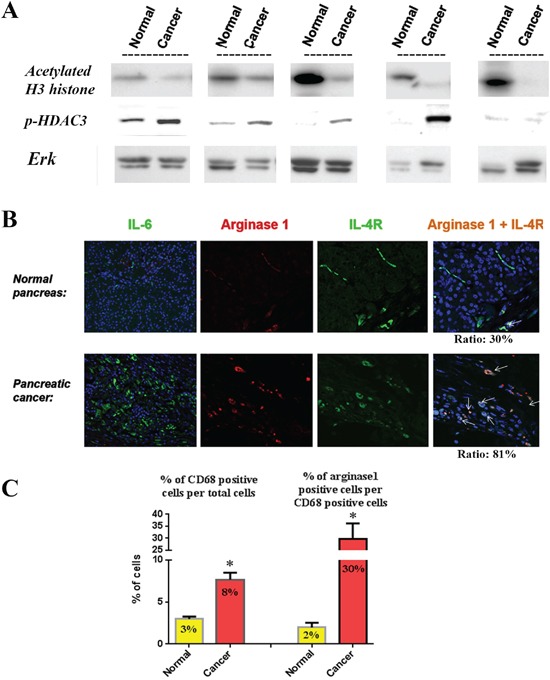
Relationship between histone acetylation, p-HDAC3, IL-6, macrophage phenotype and IL-4 receptor in human tissue samples **A.** Levels of acetylated H3 histones and p-HDAC3 were measured by Western in human tissue samples from pancreatic tumors and their matching normal-appearing tissues (from the same patient). Blots were re-probed for Erk to confirm equal loading. **B.** Immuno-fluorescence staining of IL-6, Arginase1, IL-4 receptor and dual staining for Arginase1 and IL-4 receptor in normal and cancer human pancreatic tissues. **C.** Quantification of the cells stained positive for M2 macrophages (CD68 and Arginase1) and macrophage expressing IL-4 receptor (CD68 and IL-4 receptor).

To verify the validity of our animal and *in vitro* data in humans we measured the protein level of histone acetylation in human pancreatic cancer tissue samples and their matching pancreatic normal-appearing tissues from the same patients. We found that histone acetylation was decreased in all cancer tissues compared to their matching normal tissues (Fig 7A). Similarly, protein level of p-HDAC3 was increased in 4 out of 5 human cancer tissue samples compared to their matching normal-looking tissues (Fig 7A). Furthermore, staining of pancreatic tissue samples from cancer and normal patients showed increase in the level of IL-6 cytokine, M2 macrophages, and IL-4 receptor levels on macrophages as measured by immunostaining (Fig 7B). Of note, IL-6 staining was observed in many cell types in the cancer tissue including cancer cells and stellate cells compared to almost no staining in normal tissue. The role of stellate cells in the micro-environment cell interactions is investigated in a separate study. Quantification of the number of M2 macrophages shows a significant 2.5-times increase in cancer tissue compared to normal tissue (Fig 7C). The number of M2 macrophages (dual positive for CD68 and Arginase1) was increased by 15 times in cancer tissue (30% of total macrophages) compared to normal tissue (2% of total macrophages) (Fig 7C). Furthermore, the level of IL-4 receptor staining in M2 macrophages was increased from 30% of M2 macrophages in normal tissues to 81% in cancer tissues (Fig 7B). All these data in human tissue samples confirm our findings in mice.

## DISCUSSION

In this study we have developed a model of early PDAC induced by smoking, a major risk factor for the disease. We used genetically altered animals for this model using the Pdx1-Cre;LSL-Kras mice which spontaneously develop PanIN lesions [21], the precursors of PDAC. Here we provide information in these genetically altered animals that exposure to cigarette smoke promotes these precancerous lesions through a mechanism that involves HDAC, especially p-HDAC3.

First, cigarette smoke induced a significant stimulation in the amount and the size of pancreatic lesions. Associated with neoplasia development were several other features of human PDAC including a tumor microenvironment consisting of fibrosis, activated stellate cells, and M2 macrophages along with markers of EMT. Cigarette smoke exposure induced a significant 8-fold increase in pancreatic lesions. Cigarette smoking induced an increase of 30% in fibrosis. In KC mice, the increase in collagen deposition was associated with an increase in stellate cells activation. Stellate cells are considered responsible for the large amount of collagen production in the tumor micro-environment [27-29].

Further analysis focused on the presence of the macrophages, especially M2 macrophages showed a 30% increase in their number in the KC mice exposed to cigarette smoke compared to KC mice exposed to filtered air.

We observed a general tendency of an increase in the level of M2 macrophages in mice exposed to cigarette smoke compared to control treated mice. The effect of smoking on macrophages confirms the recently published findings in a mice model of pancreatic cancer [30].

A very important finding of the study is that smoking can mediate EMT at the stage of pancreatic lesions, long before reaching the adenocarcinoma stage. Markers of EMT such as a decrease in E-cadherin and increase in vimentin were stimulated by cigarette smoke. This is crucial finding knowing the association between EMT and metastasis and cancer stemness [31, 32].

The role of HDAC and macrophages in promoting pancreatic cancer development was investigated in our study. We found that smoking decreased histone acetylation in a similar fashion. Further, we found that inhibition of HDAC prevents neoplastic lesion formation, fibrosis, and M2 macrophage in the KC mice exposed to cigarette smoke.

Our *in vitro* data showed that among the HDAC family HDAC3 mediates a critical interaction between cancer cells and macrophages leading to stimulation of the macrophage phenotype change towards the pro-cancer M2 phenotype. This effect is mediated by the IL-6 cytokine. We found that smoking compounds stimulate activation/phosphorylation and translocation of HDAC3 to the nucleus, and that pharmacological and molecular inhibitions of HDAC3 decrease the level of IL-6 produced by the cancer cells. IL-6 mediated, at least in part, the phenotypic change in macrophages through regulation of IL-4 receptor expression leading to more interaction with IL-4 in the micro-environment. The HDAC3 regulation of IL-6 in the pancreatic cancer cells and its effect on regulating the macrophage phenotype is a novel finding.

The pro-tumor M2 macrophages in turn, slightly, but significantly, stimulated survival of the pancreatic cancer cells. More importantly, we found that M2 macrophage conditioned media stimulated expression of EMT markers in pancreatic cancer cells. This effect could be mediated by TGF-β and IL-10 [11, 33].

EMT is an important mediator of early metastasis and resistance to treatments indicating development of these features of cancer aggressiveness in early stages of the disease [31, 32, 34].

The analysis of human pancreatic tissue samples confirmed our findings in animals and cells. The pancreatic cancer tissue samples were compared to their matching tissue samples isolated from the normal looking pancreatic tissue areas from the same patient. Human pancreatic cancer tissues expressed lower levels of histone acetylation, higher level of p-HDAC3 and M2 macrophage marker Arginase1, and higher levels of IL-6 and IL-4 receptor compared to normal tissue. This is the first data showing such associations in the human pancreatic cancer tissue samples.

Recently published data showed that inhibition of IL-6 prevents promotion of pancreatic cancer in mice [35]. Our data show the mechanism of regulation of IL-6 production in the context of pancreatic cancer development through a pathway that involves HDAC3 activation.

Preventing EMT is of critical importance in the fight against pancreatic cancer. In fact, chemotherapeutic agents are very successful in killing and preventing proliferation of the cancer cells; yet, EMT-mediated metastasis and cancer stemness induce a rapid spreading of the disease in the body as well as a rapid development of resistance to treatments. Therefore, our findings open a possibility to target these key components of the pathway mediating cancer cell evasion from treatments. This study shows a critical role of IL-6 in mediating the interaction between cancer cells and macrophages. However, we cannot exclude that IL-6 is also produced by macrophages and pancreatic stellate cells and may regulate other interactions, especially involving pancreatic stellate cells [36].

**Figure 8:**
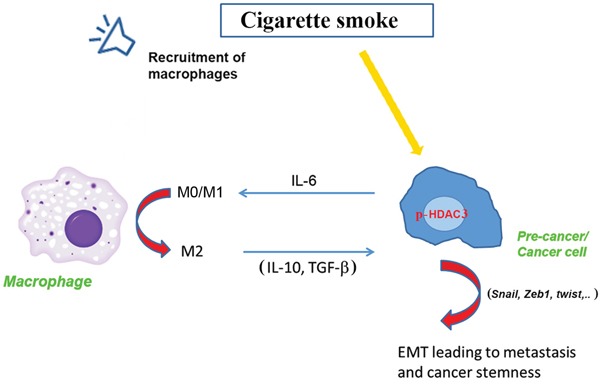
Representative scheme of the effect of smoking on p-HDAC3 leading to a cross talk between cancer cells and macrophages and promotion of the cancer

In summary, we have developed an animal model of pancreatic cancer precursors induced by a combination of a genetic mutation and exposure to cigarette smoke. An important finding of the study is the role of HDAC in promoting pancreatic cancer through a mechanism that involves stimulation of the interaction between pre-cancer or cancer cells and macrophages. This mechanism is mediated by HDAC3 and IL-6 (Fig 8). More importantly, we found that inhibition of HDAC by the FDA approved inhibitor Saha reversed the effect of smoking and decreased the level of PanIN lesions along with inhibition of development of the tumor microenvironment. These results demonstrate a novel mechanism of cancer promotion through regulation of histone acetylation. They also demonstrate a novel mechanism of regulation of the macrophages phenotype leading to a phenotype change in the cancer cells towards the mesenchymal phenotype prone of metastasis and resistance to treatments.

## EXPERIMENTAL PROCEDURES

### Reagents

NNK was purchased from ChemSyn Science Laboratories (Lenexa, KS, USA). Cigarette smoke condensate (CSC) from Murty Pharmaceuticals Inc (Lexington, KY). Alpha-smooth muscle actin (αSMA) antibody was purchased from Sigma-Aldrich (St. Louis, MO); CD45 antibody from Thermo-Scientific (Rockford, IL); Sirius Red/Collagen Staining Kit was from Chondrex (Redmond, WA); F4/80 and vimentin antibodies from AbCam (Cambrige, MA); Arginase1 from Santa Cruz Biotechnology (Santa Cruz, CA); IL-6 antibody from Novus Biological (Littleton, CO); IL-4 receptor-α antibody from Biolegend (San Diego, CA); other antibodies were from Cell signaling (Danvers, MA). IL-4 and IL-6 recombinant peptides were from Bio Vision (Milpitas, CA). M-CSF was from Prospec (East Brunswick, NJ). Saha was from Cayman (Ann Arbor, MI). All other chemicals were from Sigma Aldrich (St. Louis, MO).

### Mouse model

Mice were generated by Dr. Paul Grippo [21, 37]. Mice were housed in a temperature of (20 -/+2°C) room with a 12 hour light-dark cycle.

The Pdx1-Cre;LSL-Kras mice (8 mice per group) were exposed to cigarette smoke for 6 hours per day, 5 days per week for duration of 7 weeks. We used humidified 3R4F cigarettes (Tobacco Health Research Institute, Lexington, KY). During the first week, the concentration of nicotine in the smoke was raised every day from 30mg/m^3^ to reach 80mg/m^3^ by the end of the week to allow adaptation of the mice to the treatment. During the remaining 6 weeks mice were exposed to cigarette smoke for an average level of 80mg/m3. The concentration of nitric oxide and carbon monoxide in cigarette smoke in the chambers was monitored daily. The level of nicotine was measured weekly during the remaining 6 weeks of the treatment.

During the last 5 weeks of the exposure mice were intra-peritoneally (i.p.) injected with Saha (50mg/Kg) or saline 5 days per week [38].

After 7 weeks, mice were sacrificed 24 hours after the last exposure to cigarette smoke, pancreas collected and preserved adequately for analysis.

All of the mice were housed in Association for Assessment and Accreditation of Laboratory Animal Care-accredited facilities and used in accordance with the NIH Guide for Care and Use of Laboratory Animals.

### Luminex assay

Luminex assay was performed at Stanford Human Immune Monitoring Center as recommended by the manufacturer (Panomics/Affymetrix). Assays were performed in duplicate using the Luminex 200 IS System (Luminex Corp.) as reported previously [39]. Individual cytokines and chemokines were identified and classified by the red laser, and levels were quantified using the green laser. Digital images of the bead array were captured after laser excitation and processed on a computer workstation. Standard curves and reports of unknown samples were prepared using BeadView and MiraiBio software.

### Human pancreatic tissue samples

Human pancreatic tissue samples were received from Dr. Nissen, the Director of Hepatobiliary and Pancreatic Surgery at Cedars-Sinai Medical Center. The study is approved by Cedars-Sinai under the IRB protocols # 4201and # 34086. Informed consents were obtained from all subjects before collecting tissue specimens.

### Macrophage preparation

For Bone Marrow Derived Macrophage (BMDM) preparation, in brief, both ends of femur and tibia were cut and flushed with a syringe filled with complete RPMI 1640 containing 10% fetal bovine serum (cRPMI) to extrude BM cells into a sterile petri dish. After gentle re-suspension and centrifugation, BM cells were cultured using 20% L929 cell conditioned medium (as a source of granulocyte/macrophage colony-stimulating factor) in cRPMI. On day 4, unattached cells were discarded, and medium was replaced with a fresh batch containing the L929 cell conditioned medium as above. Cells were ready for use on day 6 [40]. For human macrophage preparation, in brief, human peripheral blood mononuclear cells (PBMCs) were isolated from buffy coats by Ficoll-Hypaque density gradient centrifugation, and then monocytes were further enriched by CD14^+^ magnetic beads (Miltenyi Biotec). Enriched monocytes were cultured with complete RPMI medium containing 50-100 ng/ml hM-CSF for 6 days. At day 7, human macrophages were ready for use [41].

### Immunohistochemistry

Samples of pancreas were fixed in formalin and paraffin embedded. Paraffin-embedded sections of pancreas were stained with hematoxylin and eosin to determine the presence of pancreatic lesions and for standard histological examination. Fibrosis was evaluated by immunostaining with Sirius Red/Collagen antibody and activated stellate cells identified by αSMA staining. Inflammatory cells in the pancreas were evaluated by immunostaining with anti-CD45 antibody. Cell proliferation was evaluated by PCNA staining.

### Immunofluorescence analysis

Immunofluorescence analysis of the pancreas after smoke exposure was performed on 5-μm-thick tissue sections mounted onto glass slides that were baked overnight at 58°C. Tissues were deparaffinized with xylene, rehydrated in decreasing concentrations of ethanol and permeabilized for 30 min with methanol solution. Antigen retrieval was performed by boiling sections in 0.5% citrate buffer for 15 min, blocked with 2.5% horse serum for 1 h and incubated with murine F4/80 and Arginase1 (Santa Cruz Biotechnology, Santa Cruz, CA) primary antibodies overnight at 4°C. Slides were washed with PBS and incubated with FITC-conjugated (A-629511, Invitrogen, Grand Island, NY, USA) and Alexa-Fluor-647 (A-21447, Invitrogen)-conjugated secondary antibodies for 1 h, washed three times and mounted with Vectashield containing 4′ 6′-diamidino-2-phenylindole (DAPI).

### Western blot

Tissue was homogenized and re-suspended in RIPA phosphorylation buffer (50 mM NaCl, 50 mM Tris/HCl pH 7.2, 1% deoxycholic acid, 1% Triton X-100, 0.1% SDS, 10 mM Na_2_HPO_4_ + NaH_2_PO_4_, 100 mM NaF, 2 mM Na_3_VO_4_, 80 μM glycerophosphate, 20% glycerol, 1 mM PMSF, 5 μg/ml each of pepstain, leupeptin, chymostatin, antipain, and aprotinin), sonicated and centrifuged for 15 min at 16,000 x g at 4°C. Proteins in the supernatant were separated by SDS-PAGE and electrophoretically transferred to nitrocellulose or PVDF membranes. Non-specific binding was blocked for 1 h with 5% bovine serum albumin or non-fat dry milk in Tris-buffered saline (4 mM Tris base, 100 mM NaCl, pH 7.5) containing 0.05% Tween 20. Membranes were incubated with primary antibody overnight at 4°C, and then for 1 h with peroxidase-conjugated secondary antibody. Blots were developed using Supersignal Chemiluminescent Substrate (Pierce, Rockford, IL).

### Cell culture and transfection

The poorly differentiated MIA PaCa-2 and moderately differentiated Bx-PC3 human pancreatic adenocarcinoma cell lines were obtained from the American Type Culture Collection (Manassas, VA). MIA PaCa-2 cells were grown in 1/1 D-MEM/F-12 medium (GIBCO Invitrogen Corporation, Grand Island, NY) supplemented with 15% fetal bovine serum (FBS), 4 mM l-glutamine, and 1% of antibiotic/antimicotic solution (Omega Scientific, Tarzana, CA). Bx-PC3 were grown in RPMI-1640 (GIBCO Invitrogen Corporation, Grand Island, NY) supplemented with 10% fetal bovine serum (FBS) and 1% of antibiotic/antimicotic solution (Omega Scientific, Tarzana, CA). Cells were maintained at 37°C in a humidified atmosphere containing 5% CO_2_ and were used between passages 2 and 10.

Transient transfections of MIA PaCa cells were performed using the electroporation Amaxa System Nucleofector^™^ (Amaxa Inc, Gaithersburg, MD) according to the manufacturer protocol. HDAC3 siRNA, (MWG biotech, High Point, NC) was applied to the cells using electroporation according to the Amaxa kit protocol. Control cells were transfected with the Silencer Negative Control siRNA #1 (Ambion; Foster City, CA).

### Flow cytometry

For surface staining, cells were stained with APC-conjugated IL-4 receptor-α (Biolegend). Cells were washed, stained with surface markers. Dead cells were excluded from analysis using violet viability stain (Invitrogen). Cells were acquired on a Cyan (Beckman Coulter, Brea, CA) and analyzed with Summit v4.3.

### Statistics

Statistical analyses of the immunohistochemical quantifications and biochemical measurements were performed by using Student's *t* test, one-way ANOVA, or Fisher's exact test with GraphPad Prism (GraphPad Software). A *p* value < 0.05 was considered statistically significant. The data showed is the result of at least 3 different experiments.
